# m6A Reader Igf2bp1 Regulates the Inflammatory Responses of Microglia by Stabilizing *Gbp11* and *Cp* mRNAs

**DOI:** 10.3389/fimmu.2022.872252

**Published:** 2022-04-29

**Authors:** Lu Ding, Huiran Wu, Yi Wang, Yun Li, Zhanping Liang, Xiaohuan Xia, Jialin C. Zheng

**Affiliations:** ^1^ Center for Translational Neurodegeneration and Regenerative Therapy, Tongji Hospital Affiliated to Tongji University School of Medicine, Shanghai, China; ^2^ Translational Research Center, Shanghai Yangzhi Rehabilitation Hospital Affiliated to Tongji University School of Medicine, Shanghai, China; ^3^ Translational Research Institute of Brain and Brain-Like Intelligence, Shanghai Fourth People’s Hospital Affiliated to Tongji University School of Medicine, Shanghai, China; ^4^ Shanghai Frontiers Science Center of Nanocatalytic Medicine, Tongji University, Shanghai, China; ^5^ Collaborative Innovation Center for Brain Science, Tongji University, Shanghai, China

**Keywords:** Igf2bp1, Gbp11, Cp, m6A reader, microglial activation, neuroinflammation

## Abstract

Microglia are brain resident cells that function as brain phagocytic macrophages. The inflammatory responses of microglia induced by pathologic insults are key regulators in the progression of various neurological disorders. Currently, little is known about how these responses are regulated intrinsically. Here, it is observed that LPS-activated microglia exhibit distinct N6-methyladenosine (m6A) methylation patterns that are positively correlated with the expression patterns of corresponding mRNAs. High-throughput analyses and molecular studies both identified Igf2bp1 as the most significantly regulated m6A modifiers in activated microglia. Perturbation of function approaches further indicated Igf2bp1 as a key mediator for LPS-induced m6A modification and microglial activation presumably *via* enhancing the m6A methylation and stability of *Gbp11* and *Cp* mRNAs. Thus, our study provides a possible mechanism for the m6A methylation-mediated microglia regulation and identifies Igf2bp1 as a potential target for modulating the inflammatory responses of microglia.

## Introduction

Microglia are brain resident cells that account for approximately 10% of total cells (1, 2). They function as brain phagocytic macrophages that regulate brain development, normal brain function, immune responses, and injury repair (1, 2). Microglia are activated by pathologic insults in the brain such as microbes, dead cells, and protein aggregates (e.g., Aβ, phospho-Tau) (3). Although microglial activation is a highly dynamic process, activated microglia can be roughly classified into two main phenotypes, including the pro-inflammatory M1 phenotype (classical activation), and the anti-inflammatory M2 phenotype (alternative activation) (1, 3). Emerging evidence has implicated the activation of microglia, especially that of pro-inflammatory ones, as either a causal factor or an important contributor of the progression of both acute brain injury (e.g., stroke, traumatic brain injury) and chronic neurodegenerative diseases (e.g., Alzheimer’s disease, Multiple sclerosis) (1, 4). Pro-inflammatory microglia exhibit reduced phagocytic capacity but enhanced pro-inflammatory molecules (e.g., cytokines and chemokines) production ability, resulting in neurotoxicity (1, 4).

The transition of microglial pro-inflammatory phenotype is under strict regulation extra- and intra-cellularly (3, 4). Recent studies have implicated RNA modifications as one key intracellular mechanism for the activation of microglia in response to extracellular stimuli (5). Among all types of RNA modifications, N6-methyladenosine (m6A) RNA methylation is the predominant one with most extensive investigations, which occupies approximately 0.3% of total adenosine residues (6, 7). The m6A modification affects various RNA metabolisms, including RNA processing, nuclear export, RNA translation to decay (6). m6A is reversibly and dynamically regulated by m6A methyl-transferase (writers), demethylases (erasers), and m6A binding proteins (readers) (6). It has been reported that the expression of m6A writer, Mettl3, can be induced by lipopolysaccharide (LPS), which participates in microglial activation regulation (7). However, the roles of other m6A modifiers especially m6A readers that recognize, bind to, and stabilize m6A methylated mRNAs to enhance translation remain largely unknown (6, 8). In this study, we determined the shift of m6A signatures of LPS-induced microglia and their correlation with the RNA expression patterns. We also identified insulin-like growth factor 2 mRNA binding protein 1 (Igf2bp1, also known as Imp1), a recently discovered m6A reader, as the most significantly regulated m6A regulator post LPS-treatment. The perturbation of function approaches revealed Igf2bp1 as an important protein that mediates the inflammatory responses and the shift of m6A signatures of LPS-induced microglia. At last, we demonstrated that Igf2bp1 mediates microglial activation presumably *via* stabilizing its target mRNAs *Gbp11* and *Cp.*

## Materials and Methods

### Mice and Microglia Culture

C57BL/6 mice were housed and bred in the Comparative Medicine animal facilities of Tongji University School of Medicine (TUSM). All procedures were conducted according to protocols approved by the Institutional Animal Care and Use Committee (IACUC) of TUSM. Mouse primary microglia were isolated from whole brains of C57 mice at postnatal day 1 as previously described (9). Briefly, neonatal mouse brains were dissected out after removing peripheral blood vessels and washed with HBSS. Mouse brains were digested at 37°C for 30 min in 0.25% trypsin solution (Invitrogen) supplemented with 0.05% DNase I (Invitrogen). Digestion was stopped by FBS (Invitrogen). The tissue dissociates were centrifuged at 1500 rpm for 5 min at 4°C. Dissociated cells were re-suspended and cultured in DMEM with 10 ng/mL GM-CSF, 10% FBS, 50 U penicillin, and 50 mg/mL streptomycin at 37°C. The culture medium was replaced every three days. Microglia in the microglia-astrocytes mixed cultures were induced to detach by shaking and collected by 1500 rpm centrifugation for 5 min at 4°C.

### Transfection of siRNA

The siRNA scrambled control and siRNAs for Igf2bp1 (sense sequence: 5’-GUCCCAAGGAGGAAGUAAATT-3’), Gbp11 (sense sequence: 5’-GGCCUUAUUUCAUUCUUUATT-3’), and Cp (sense sequence: 5’-GCCACCAAUUCAUGCAAAUTT-3’) were purchased from GenePharma (GenePharma Co., Ltd, Shanghai). Transfection was performed using the HiPerFect Transfection Reagent (Qiagen301705) according to the manufacturer’s instruction. Briefly, microglia were seeded at 1 × 10^6^ cells per well of a 6-well plate in 1500 µl of microglia culture medium one day before transfection. 3 μg siRNA was diluted in 1 ml culture medium without FBS. After 5 minutes, 12 µl HiPerFect Transfection Reagent was then add to the diluted siRNA. siRNA and transfection reagent were incubated for 10 min at room temperature to allow formation of transfection complexes. The complexes were added drop-wise onto the cells. After 8 h, siRNA-containing medium was replaced with microglia culture medium. Transfection efficiency was determined by examining the transcript levels of target genes 48 h post transfection *via* qRT-PCR. Specific primer sets were included in **Supplementary Table 1**.

### Quantitative Reverse Transcription Polymerase Chain Reaction (qRT-PCR)

The messenger RNAs (mRNAs) were isolated from cell samples using Rneasy mini kit (Qiagen) according to the manufacturer’s instructions. Genomic DNA was removed using Dnase I digestion kit (Qiagen) and cDNA was synthesized using miScript II RT kit (Qiagen). Transcripts were amplified using specific primer sets (**Supplementary Table 1**) and SYBR green PCR kit (Qiagen) with the ABI7500 (Applied Biosystems). Reactions were run in triplicates for each sample and no-template blanks were used as negative controls. *Gapdh* was used as internal control for value normalization.

### Western Blotting

Western blotting was performed as previously described (9). Cell samples were lysed in RIPA lysis and extraction buffer (ThermoFisher) containing a protease inhibitor cocktail (Sigma). Protein concentrations were determined using BCA Protein Assay Kit (Pierce). Proteins (20~30 mg) from cell lysates were separated by sodium dodecyl sulfate polyacrylamide gel electrophoresis (SDS-PAGE) and electrophoretic transferred to polyvinylidene fluoride membranes (Millipore and Bio-Rad). Membranes were incubated with primary antibodies for TNFα (rabbit, Abcam, ab183218, 1:1000), IL1β (rabbit, Abcam, ab234437, 1:1000), CD68 (mouse, BD Abcam, ab125212, 1:1000), and β-actin (mouse, Abcam, ab8226, 1:10000), overnight at 4°C followed by a secondary anti-rabbit or anti-mouse antibody (Cell Signaling Technologies, 1:10000) incubation. Antigen-antibody complexes were visualized by Pierce ECL Western Blotting Substrate (ThermoFisher). Membrane images were acquired using CanonScan 9950F scanner and analyzed using ImageJ program.

### Enzyme-Linked Immunosorbent Assay

Conditioned medium of primary mouse microglia with/without the perturbation of gene function was collected and the concentration of pro-inflammatory cytokines were measured with commercially available ELISA kits (Multi Sciences, EK282/4) according to manufacturer’s protocols. Briefly, microglia were seeded onto the 6-well at a density of 1 × 10^6^ cells/well. 48 h after siRNA transfection, culture medium was collected and centrifuged at 300 g for 10 min. Diluted standards and culture medium in triplicate were added to the corresponding wells and incubated at room temperature for 2 hours on a microplate shaker. Sample Diluent was used as blank control. 100 µl Diluted Streptavidin-HRP was added to all wells and incubate at room temperature for 45 minutes. 100 µl TMB Substrate Solution was then added to all wells and incubate at room temperature for 30 minutes. The enzyme reaction was stopped by quickly pipetting 100 µl of Stop Solution into each well. Absorbance of each well was read using spectrophotometer DV8200 (Drawell). TNFα concentrations were calculated according to the standard curve.

### Methylated RNA Immunoprecipitation Sequencing, RNA Sequencing, and Bioinformatics Analysis

100 μg of total RNA was extracted and purified using RiboMinus™ Eukaryote Kit v2 (A15020, Invitrogen) to deplete the ribosomal RNA from the total RNA. Next, RNA Fragmentation Reagents (AM8740, Invitrogen) were used to shear the RNA into approximately 100-nt fragments. Approximately 1/10 of the fragmented RNA was saved as the input control for further RNA sequencing. The remaining were incubated with an anti-m6A antibody (202,203, Synaptic Systems) for one hour at 4°C, and then mixed with prewashed Pierce™ Protein A/G Magnetic Beads (88,803, Thermo Scientific) in immunoprecipitation buffer at 4°C overnight. The m6A antibody was digested with proteinase K digestion buffer and the methylated RNA was purified for MeRIP-seq. Paired-end 2 × 150 bp sequencing was performed on the Illumina Novaseq 6000 platform of LC-BIO Bio-tech. (Hangzhou, China). Mapped reads of immunoprecipitation and input libraries were provided for R package exomePeak. HOMER was used for *de novo* and known motif finding, followed by localization of the motif with respect to peak summit by Perl scripts. Then, StringTie was used to measure expression levels for all mRNAs from input libraries by calculating fragments per kilobase of transcript per million fragments mapped (FPKM). Differential expression analysis was performed using the DESeq2 R package. P-values and q-values were adjusted using the Benjamini and Hochberg’s approach for controlling the false discovery rate. Genes with q-value < 0.05 were considered as differentially expressed. Differentially expressed genes (DEGs) with over 2 fold changes were mapped to Gene ontology (GO) and Kyoto Encyclopedia of Genes and Genomes (KEGG) pathways analysis. GO and KEGG enrichment analysis was performed using The Database for Annotation, Visualization and Integrated Discovery (DAVID) (http://david.ncifcrf.gov/).

### RNA Stability Assay

To detect objective RNA stability, primary microglia were seeded in 12-well plates and treated with 5 μg/mL actinomycin D (Med-ChemExpress) and then collected at the indicated time points. Total RNA was extracted by RNeasy mini kit (Qiagen) according to the manufacturer’s instructions. qRT-PCR assay was used to determine the levels of mRNAs. Specific primer sets were included in **Supplementary Table 1**. *Gapdh* was used as internal control for value normalization. The mRNA half-live times were estimated according to the linear regression analysis.

### Statistical Analyses

All results are the means of at least three independent experiments ± s.d. Data from two groups were evaluated statistically by two-tailed unpaired student *t* test. Data from multiple groups were evaluated statistically by either one-way ANOVA followed by Tukey’s *post hoc* test or two-way ANOVA followed by Sidak’s multiple comparisons test according to the experiment design. Significance was considered when *p* < 0.05.

## Results

### Pro-Inflammatory Microglia Exhibit Positive Correlation Between m6A Methylations and Gene Expression Profiles

To examine the changes of m6A profiles in microglial activation, we utilized LPS to stimulate the inflammatory responses of microglia *in vitro* (9). Mouse primary microglia were treated with 100 ng/ml LPS for 3 h and the inflammatory responses of microglia were tested at 48 h. Western blotting results found enhanced expression of pro-inflammatory proteins TNFα, IL1β, and CD68 in LPS-treated microglia (**Supplementary Figure 1A**). The expression of transcripts corresponding to pro-inflammatory genes *TNF*, *Il1b*, and *Nos2* was similarly increased in LPS-treated microglia (**Supplementary Figure 1B**). The release of TNFα from LPS-treated microglia was also promoted, confirming the successful activation of microglia *via* LPS stimulation (**Supplementary Figure 1C**).

**Figure 1:**
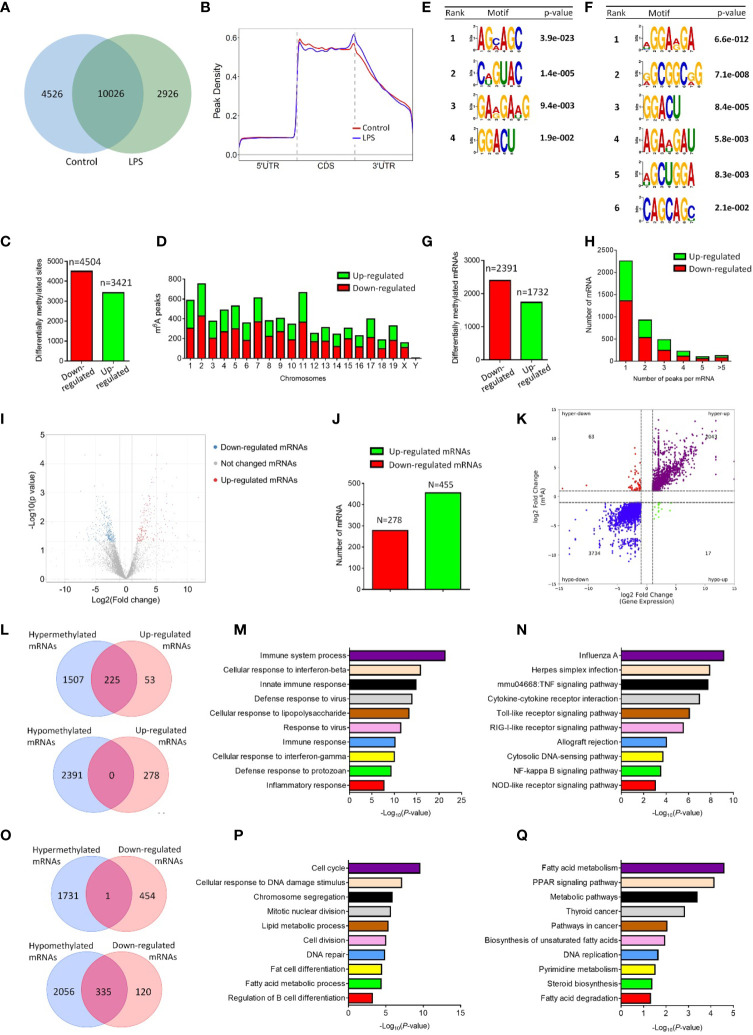
LPS stimulation alters the m6A modification patterns of primary microglia. **(A)** Venn diagram showing the detected m6A peaks in control and LPS-stimulated microglia. **(B)** Accumulation of the region of average m6A peaks along all transcripts in control and LPS-stimulated microglia. **(C)** The numbers of significantly altered m6A peaks after LPS treatment. **(D)** The distributions of altered m6A peaks in human chromosomes. **(E, F)** The m6A motifs enriched from the detected m6A peaks in control **(E)** and LPS-stimulated microglia **(F)**. **(G)** The numbers of mRNAs with significantly altered m6A peaks after LPS treatment. **(H)** The distribution of altered m6A peaks per mRNA. **(I)** Volcano plots showing the differentially expressed genes in microglia post LPS-stimulation. **(J)** The numbers of differentially expressed genes in microglia post LPS-stimulation. **(K)** Four-quadrant plots showing the distribution of genes with altered m6A modification and mRNA levels. **(L)** Venn diagrams for genes with either up-regulated expression and m6A hypermethylation (upper panel) or up-regulated expression and m6A hypomethylation (lower panel). **(M)** The top 10 GO terms of genes with m6A hypermethylation and up-regulated expression. **(N)** The top 10 KEGG pathways of genes with m6A hypermethylation and up-regulated expression. **(O)** Venn diagrams for genes with either down-regulated expression and m6A hypermethylation (upper panel) or down-regulated expression and m6A hypomethylation (lower panel). **(P)** The top 10 GO terms of genes with m6A hypomethylation and down-regulated expression. **(Q)** The top 10 KEGG pathways of genes with m6A hypomethylation and down-regulated expression.

The m6A signature of pro-inflammatory microglia was then determined by MeRIP-seq approach. MeRIP-seq analysis identified 12952 and 14552 m6A sites in control and LPS-stimulated microglia, respectively, and among them, 10026 m6A sites were shared by both groups (**Figure 1A**). MeRIP-seq analysis showed that m6A peaks both in control and LPS-stimulated microglia were primarily enriched in the coding sequence and 3’ untranslated regions (UTR) regions (**Figure 1B**). Furthermore, there were 3421 up-regulated m6A peaks and 4504 down-regulated m6A peaks in LPS-stimulated microglia, which distributed in all chromosomes (**Figures 1C, D**). The sequence logo showed distinct enriched m6A motifs in control and LPS-stimulated microglia (**Figures 1E, F**). By analyzing the localization of all m6A sites in mRNAs, 2391 and 1731 mRNAs were identified with down-regulated and up-regulated m6A modifications, respectively (**Figure 1G**). The majority of mRNAs had one m6A peak (about 55%) (**Figure 1H**). Meanwhile, there were over 3% of mRNAs had over 5 m6A peaks (126/4123) (**Figure 1H**).

To examine the association of m6A methylation with gene expression in activated microglia, we then carried out RNA-seq using control and LPS-treated microglia. 455 and 278 mRNAs were significantly up- and down-regulated in LPS-treated microglia, respectively, versus controls (**Figures 1I, J**). Afterwards, four-quadrant plots were utilized to divide mRNAs with over two folds m6A methylation and expression levels between control and LPS-treated microglia (**Figure 1K**). Among them, 2106 hypermethylated mRNAs that were significantly up-regulated (2043) or down-regulated (63) in expression, and 3751 hypomethylated m6A peaks in mRNAs that were significantly up-regulated (17) or down-regulated (3734) in expression (**Figure 1K**). We next drew venn diagram using miRNAs with significantly different m6A methylation and expression levels (over two folds, p<0.05). Over 80% of up-regulated mRNAs were hypermethylated (**Figure 1L**), and about 75% of down-regulated mRNAs were hypomethylated (**Figure 1O**). In contrast, only 1 down-regulated mRNA was hypermethylated (**Figure 1O**), and no overlapping mRNAs were identified among up-regulated and hypomethylated mRNAs (**Figure 1L**). We next examined the potential roles of mRNAs with altered m6A methylation and expression patterns *via* GO and KEGG analyses. GO biological process analysis revealed that the identified 225 mRNAs with up-regulated m6A peaks and expression levels were enriched in immune response-related GO terms, suggesting the strong association of m6A-hypermethylated mRNAs with the pro-inflammatory function of microglia (**Figure 1M**). Similarly, KEGG analysis indicated that the mRNAs with up-regulated m6A peaks and expression levels were enriched in inflammatory pathways including TNFβ, Toll-like receptor, and Nf-κb signaling (**Figure 1N**). In contrast, both GO and KEGG analyses revealed that the mRNAs with down-regulated m6A peaks and expression levels were involved in cell cycle and metabolic regulation (**Figures 1P, Q**). Hence, the MeRIP-seq and RNA-seq results demonstrated a positive correlation between the m6A methylation and expression of pro-inflammatory genes in LPS-stimulated microglia, implying the involvement of m6A modification in the regulation of microglial activation.

### Igf2bp1 is the Most Significantly Regulated m6A Regulator

**Figure 2:**
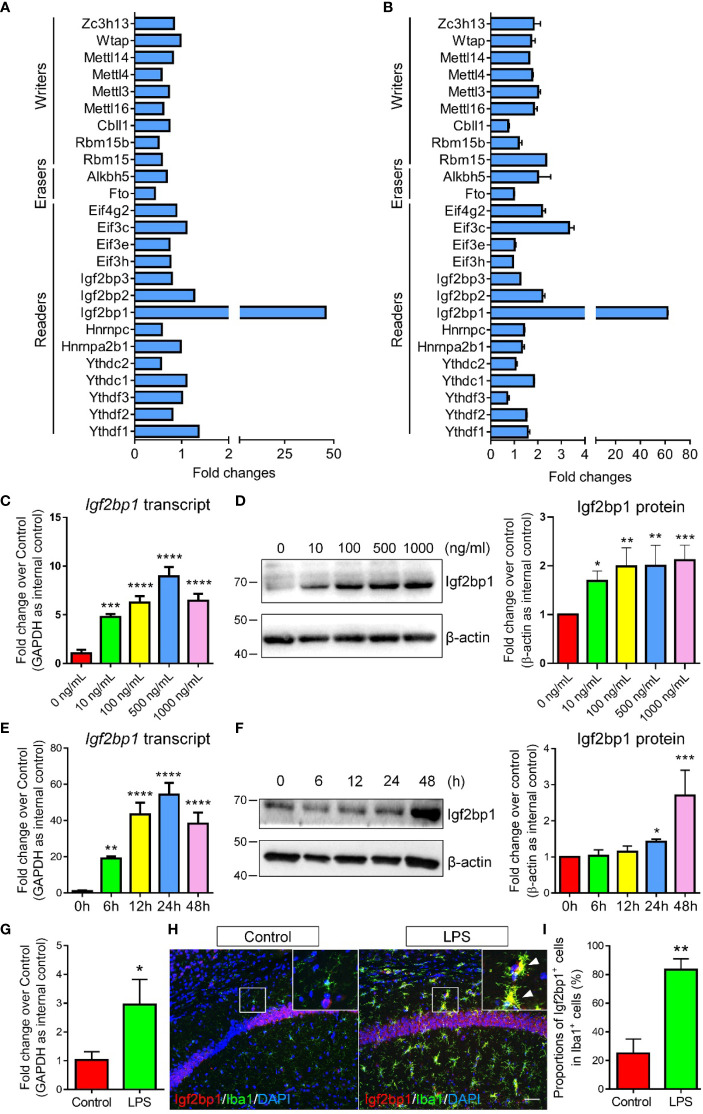
Igf2bp1 expression is induced by LPS treatment. **(A)** RNA-seq results showing the expression patterns of m6A modifiers. **(B)** qRT-PCR results showing the expression patterns of m6A modifiers. **(C)** *Igf2bp1* mRNA expression in microglia after LPS treatment at indicated doses. **(D)** Igf2bp1 protein expression in microglia after LPS treatment at indicated doses. **(E)** *Igf2bp1* mRNA expression in microglia after LPS treatment at indicated time points. **(F)** Igf2bp1 protein expression in microglia after LPS treatment at indicated time points. **(G)** *Igf2bp1* mRNA expression in mouse hippocampal tissues in control and LPS-injection groups. **(H)** Co-expression of Igf2bp1 and Iba1 in mouse hippocampal tissues in control and LPS-injection groups. Insets represent high-magnification images of the corresponding small box area. Arrows point cells displaying Igf2bp1 and Iba1 immunoreactivities. **(I)** Quantification of the numbers of Igf2bp1^+^ cells in Iba1^+^ activated microglia in **(H)**. Scale bar: 50 μm. Data were represented as mean ± s.d. from three independent experiments. *, **, ***, and **** denote *p* < 0.05, *p* < 0.01, *p* < 0.001, and *p* < 0.0001, respectively.

To identify the key factor that mediates the LPS-induced m6A modifications, we first screened the expression patterns of currently known m6A writers, erasers, and readers in control and LPS-treated microglia using RNA-seq data (**Figure 2A**). Among all tested m6A regulators, *Igf2bp1*, a recently identified m6A reader (10, 11), was demonstrated as the most significantly regulated one. The RNA-seq data was confirmed by qRT-PCR assay, in which the expression levels of *Igf2bp1* transcripts exhibited the largest fold changes in LPS-treated microglia versus controls (**Figure 2B**). Furthermore, we determined the effects of LPS stimulation on Igf2bp1 expression by treating microglia with different doses of LPS. qRT-PCR results revealed an elevation of *Igf2bp1* transcript levels with the increase of LPS concentration till 500 ng/ml (**Figure 2C**). Western blotting results suggested that the expression of Igf2bp1 proteins was promoted by increasing LPS concentration from 0 ng/ml to 100 ng/ml (**Figure 2D**). No significant difference was observed in the levels of Igf2bp1 proteins when microglia were treated with LPS of 100 ng/ml or higher doses (**Figure 2D**). In addition, the expression levels of both *Igf2bp1* mRNAs and Igf2bp1 proteins were also elevated by LPS treated in a time-dependent manner (**Figures 2E, F**).

Next, we examined the expression levels of Igf2bp1 in mouse brains under inflammatory conditions. Neuroinflammation was stimulated by intraperitoneal injection of LPS for 3 d (one injection per day, dose: 1 mg/kg). qRT-PCR analysis demonstrated significant increase of *Igf2bp1* transcripts in mouse hippocampal tissues 3 days post LPS administration (**Figure 2G**). Immunohistochemical analysis further revealed a significant increase of the proportions of Igf2bp1^+^cells in Iba1^+^ activated microglia in mouse hippocampal tissues (**Figures 2H, I**). Hence, both *in vitro* and *in vivo* studies indicated that the expression of Igf2bp1 in activated microglia was significantly up-regulated, implying a role of Igf2bp1 in LPS-induced m6A modifications in microglia.

### Igf2bp1 Mediates the Inflammatory Responses and m6A Modifications of Microglia

**Figure 3:**
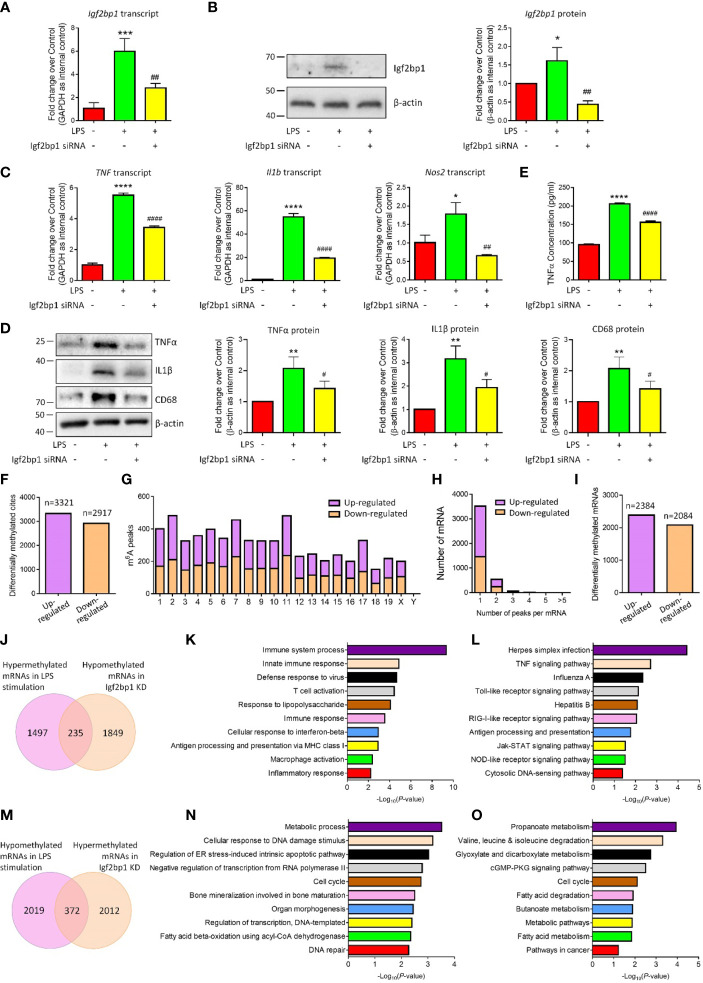
Igf2bp1 regulates the inflammatory responses and m6A modifications of microglia. **(A)** Expression of *Igf2bp1* mRNAs in LPS-stimulated microglia after knocking down Igf2bp1 expression was determined by qRT-PCR. **(B)** Expression of Igf2bp1 proteins in LPS-stimulated microglia after knocking down Igf2bp1 expression was determined by western blotting. **(C)** Expression of *TNF*, *Il1b*, and *Nos2* mRNAs in LPS-stimulated microglia after knocking down Igf2bp1 expression was determined by qRT-PCR. **(D)** Expression of TNFα, IL1β, and CD68 proteins in LPS-stimulated microglia after knocking down Igf2bp1 expression was determined by western blotting. **(E)** The release of TNFα from LPS-stimulated microglia after knocking down Igf2bp1 expression was determined by ELISA assay. **(F)** The numbers of significantly altered m6A peaks after knocking down Igf2bp1 expression in LPS-stimulated microglia. **(G)** The distributions of altered m6A peaks in human chromosomes. **(H)** The distribution of altered m6A peaks per mRNA. **(I)** The numbers of mRNAs with significantly altered m6A peaks after knocking down Igf2bp1 expression in LPS-stimulated microglia. **(J)** Venn diagrams for genes with m6A hypermethylation after LPS treatment and m6A hypomethylation post Igf2bp1 LOF. **(K)** The top 10 GO terms of genes with m6A hypermethylation after LPS treatment and m6A hypomethylation post Igf2bp1 LOF. **(L)** The top 10 KEGG pathways of genes with m6A hypermethylation after LPS treatment and m6A hypomethylation post Igf2bp1 LOF. **(M)** Venn diagrams for genes with m6A hypomethylation after LPS treatment and m6A hypermethylation post Igf2bp1 LOF. **(N)** The top 10 GO terms of genes with m6A hypomethylation after LPS treatment and m6A hypermethylation post Igf2bp1 LOF. **(O)** The top 10 KEGG pathways of genes with m6A hypomethylation after LPS treatment and m6A hypermethylation post Igf2bp1 LOF. Data were represented as mean ± s.d. from three independent experiments. *, **, ***, and **** denote *p* < 0.05, *p* < 0.01, *p* < 0.001, and *p* < 0.0001, respectively, in comparison with control microglia. ^#, ##,^ and ^####^ denote *p* < 0.05, *p* < 0.01, and *p* < 0.0001, respectively, in comparison with LPS-stimulated microglia.

To examine the effects of Igf2bp1 in the inflammatory responses of microglia, we performed loss-of-function (LOF) approaches *in vitro*. Microglia were pre-treated with 100 ng/ml LPS for 3 h and transfected with either Igf2bp1 siRNA or scrambled control for 48 h. The transfection efficiency was determined by demonstrating a ~60% reduction of *Igf2bp1* transcripts levels (**Figure 3A**) and over 70% decline of Igf2bp1 protein levels (**Figure 3B**) in LOF group versus LPS controls. qRT-PCR analysis further revealed that Igf2bp1 LOF abrogated the overproduction of transcripts corresponding to *TNF*, *Il1b*, and *Nos2* induced by LPS treatment in microglia (**Figure 3C**). Similarly, Western blotting results demonstrated that Igf2bp1 LOF significantly reduced the LPS-induced excessive expression of pro-inflammatory factors TNFα, IL1β, and CD68 (**Figure 3D**). ELISA assay also showed a significant decrease of the concentration of TNFα in the conditioned medium in Igf2bp2 LOF group, compared with LPS group (**Figure 3E**). Hence, our results suggested Igf2bp1 as a key regulator for the inflammatory responses of microglia.

Afterwards, we examined the roles of Igf2bp1 on LPS-induced m6A modification in microglia. MeRIP-seq analysis identified 3321 hypermethylated and 2917 hypomethylated m6A peaks in Igf2bp1 LOF group versus LPS group (**Figure 3F**). The differentially methylated m6A peaks distributed into all chromosomes except Y chromosome (**Figure 3G**). There were 2384 hypermethylated mRNAs and 2084 hypomethylated ones, and among them, over 80% mRNAs had one hypermethylated peak (**Figures 3H, I**). Moreover, there were 235 mRNAs that were hypermethylated with LPS stimulation and hypomethylated post Igf2bp1 LOF (**Figure 3J**). The GO and KEGG analyses revealed that those overlapping mRNAs were strongly associated with the immune responses of microglia (**Figures 3K, L**). The bioinformatical analyses indicated that Igf2bp1 LOF reversed the hypermethylation of inflammation-related mRNAs. Besides, there were 372 mRNAs that were hypomethylated with LPS stimulation and hypermethylated post Igf2bp1 LOF (**Figure 3M**). GO and KEGG analyses further demonstrated that Igf2bp1 LOF erased the LPS-induced hypomethylation of cell cycle and metabolic regulation-related mRNAs (**Figures 3N, O**). Therefore, our MeRIP-seq results showed that Igf2bp1 played an important role in the LPS-induced alteration of m6A modifications.

### Igf2bp1 Regulates Microglial Activation Presumably *via* Stabilizing *Gbp11* and *Cp* mRNAs

**Figure 4:**
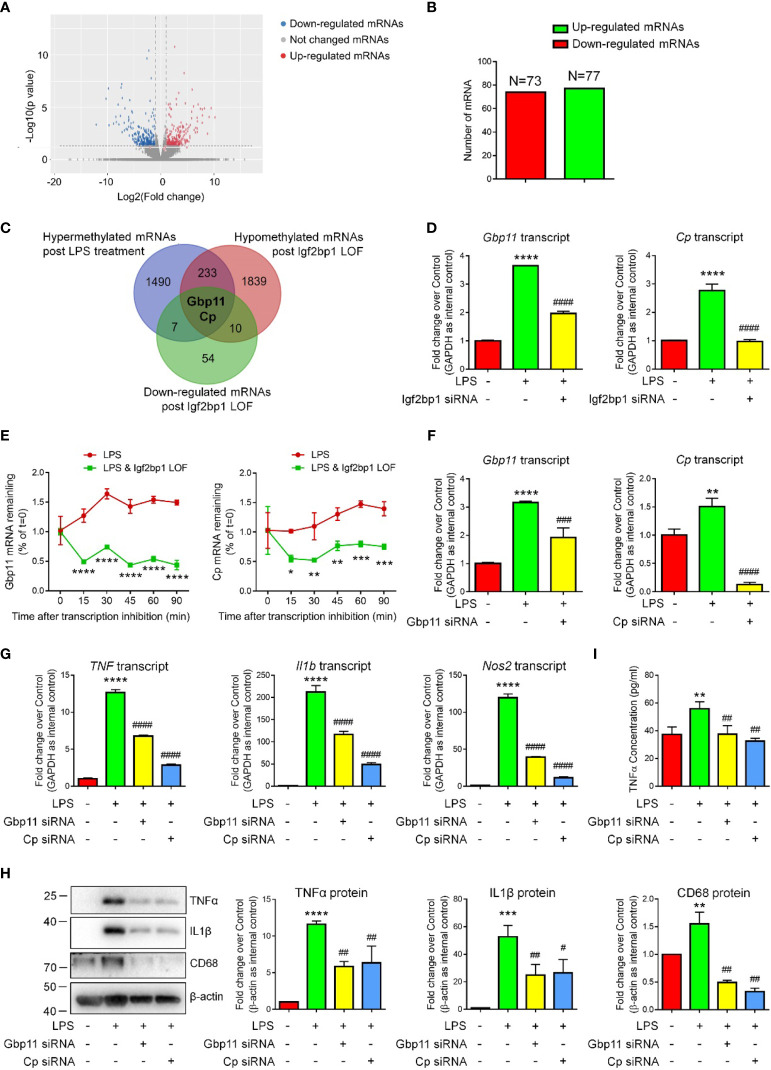
Igf2bp1 regulates the inflammatory responses of microglia *via* stabilizing *Gbp11* and *Cp* mRNAs. **(A)** Volcano plots showing the differentially expressed genes after knocking down Igf2bp1 expression in LPS-stimulated microglia. **(B)** The numbers of differentially expressed genes. **(C)** Venn diagrams for genes with m6A hypermethylation after LPS treatment, m6A hypomethylation post Igf2bp1 LOF, and reduced expression post Igf2bp1 LOF. **(D)** Expression of *Gbp11* and *Cp* mRNAs in LPS-stimulated microglia after knocking down Igf2bp1 expression was determined by qRT-PCR. **(E)** *Gbp11* and *Cp* mRNAs degradation in microglia treated with actinomycin D for the indicated times. **(F)** The knockdown efficiency of both Gbp11 and Cp siRNA in LPS-stimulated microglia was determined by qRT-PCR. **(G)** Expression of *TNF*, *Il1b*, and *Nos2* mRNAs after knocking down either Gbp11 or Cp expression in LPS-stimulated microglia was determined by qRT-PCR. **(H)** Expression of TNFα, IL1β, and CD68 proteins after knocking down either Gbp11 or Cp expression in LPS-stimulated microglia was determined by western blotting. **(I)** The release of TNFα after knocking down either Gbp11 or Cp expression from LPS-stimulated microglia was determined by ELISA assay. Data were represented as mean ± s.d. from three independent experiments.*, **, ***, and **** denote *p* < 0.05, *p* < 0.01, *p* < 0.001, and *p* < 0.0001, respectively, in comparison with control microglia. ^#, ##, ###^, and ^####^ denote *p* < 0.05, *p* < 0.01, *p* < 0.001, and *p* < 0.0001, respectively, in comparison with LPS-stimulated microglia.

Due to the importance of Igf2bp1 in microglial activation and inflammation-related m6A modification, we hypothesized that Igf2bp1 regulates the inflammatory responses of microglia *via* modifying mRNA m6A signatures. To test our premise, we first carried out RNA-seq analysis to determine the transcript expression patterns of LPS-stimulated microglia with/without Igf2bp1 LOF (**Figure 4A**). RNA-seq analysis identified 77 up-regulated and 73 down-regulated genes in Igf2bp1 LOF group versus control LPS group (**Figure 4B**). Furthermore, from the 235 genes that were hypermethylated with LPS stimulation and hypomethylated post Igf2bp1 LOF, Ceruloplasmin (Cp) and Guanylate-binding protein 11 (Gbp11) were identified with significantly reduced expression levels post Igf2bp1 LOF (**Figure 4C**). The high-throughput analysis results were confirmed by qRT-PCR which showed significant reduction of the expression levels of both *Gbp11* and *Cp* transcripts in Igf2bp1 LOF groups versus LPS controls (**Figure 4D**). To explore whether Igf2bp1 had impact on the stability of *Gbp11* and *Cp* mRNAs, we treated microglia with actinomycin D in indicated time points before total RNA was acquired. The results showed a significantly shortened half-life of both *Gbp11* and *Cp* mRNAs in Igf2bp1 deficient microglia versus control LPS group (**Figure 4E**).

To examine whether Gbp11 and Cp are potential downstream factors of Igf2bp1, we carried out Gbp11 and Cp LOF by transfecting LPS-stimulated microglia with either scrambled siRNA control, Gbp11 siRNA, or Cp siRNA. qRT-PCR results demonstrated over 50% knockdown of *Gbp11* and more than 80% knockdown of *Cp* transcripts in LOF groups versus LPS controls, suggesting efficient transfection (**Figure 4F**). qRT-PCR analysis further showed significant decline of the expression levels of transcripts corresponding to *TNF*, *Il1b*, and *Nos2* in both Gbp11 and Cp LOF groups versus LPS controls (**Figure 4G**). Western blotting results also demonstrated that both Gbp11 and Cp LOF significantly reduced the LPS-induced up-regulation of TNFα, IL1β, and CD68 protein expressions (**Figure 4H**). Moreover, ELISA assay suggested that both Gbp11 and Cp LOF erased the LPS-induced excessive release of TNFα from microglia (**Figure 4I**). Hence, our results suggested Gbp11 and Cp as pro-inflammatory proteins, implicating that Igf2bp1 mediates microglial activation *via* stabilizing *Gbp11* and *Cp* mRNAs.

## Discussion

With rapid expansion of our knowledge, m6A methylation has shown various crucial roles in the brain, including neural development, neural function maintenance, and glioblastoma tumorigenesis (12–14). Emerging evidence has suggested the involvement of m6A modification in the regulation of microglial activation. *In vitro* study revealed altered m6A modification in cytokine-treated microglia (5). It is reported that m6A writer Mettl3 enhanced microglial activation and reader Ythdc1 reduced microglial M1 polarization (7, 15). Although Nf-κb and Sirt1-related pathways have been identified as down-stream targets of Mettl3 and Ythdc1, whether these m6A modifiers regulate microglial phenotype transition through m6A modification remains unknown. In our study, we found that LPS stimulation significantly changed the m6A modification patterns of microglia, which was correlated with the mRNA expression patterns. Next, we identified Igf2bp1 as the most significantly regulated m6A modifier under LPS stimulation. Moreover, Igf2bp1 controlled the inflammatory responses of microglia and the LPS-induced m6A modification alteration. High-throughput analyses and ActD assay further identified Gbp11 and Cp as the two mRNAs whose expression levels, mRNA stability, and m6A signatures were equally regulated by Igf2bp1. At last, Gbp11 and Cp LOF significantly reduced LPS-induced microglial activation, implying that Igf2bp1 enhanced the inflammatory responses of microglia *via* enhancing the stability of Gbp11 and Cp mRNAs. Overall, we for the first time demonstrated the influences of m6A modifier on the global m6A modification patterns of microglia and its association with the regulation of microglial function. More importantly, our study implicated a key role of Igf2bp1 in microglial M1 polarization, providing a novel mechanism for microglial activation regulation.

Igf2bps, including Igf2bp1, Igf2bp2, and Igf2bp3, are RNA binding proteins that were originally considered as pro-tumorigenic proteins and stem cell advocates in the brain (16, 17). In 2018, Igf2bps were identified as m6A-binding proteins to enhance mRNA stability and translation using HEK293T cells (18). To date, our knowledge for the effects of Igf2bps as m6A readers in the brain is almost blank, except for a bioinformatics study that found Igf2bp2 up-regulation in Alzheimer’s disease patient brain samples and identified multiple predicted targets of Igf2bp2 (19). Hence, our study is the first one that performed experiment-based investigations on the participation of Igf2bps in m6A modification in brain cells. Our results suggested Igf2bp1 as the only one in Igf2bps family whose expression is robustly enhanced in activated microglia. Interestingly, Igf2bp1 and Igf2bp3 have been found to express at negligible levels in adult mouse brains (20, 21). Our results matched with these observations that Igf2bp1 is with insignificant expression in resting primary microglia, which is the reason why we pre-treated microglia with LPS and conducted Igf2bp1 LOF afterwards. In contrast, Igf2bp2 was reported to express in adult mouse brains (20, 21). Additionally, Wang et al. reported that Igf2bp2 regulates the activation of Bone marrow-derived macrophages (22). We indeed detected *Igf2bp2* mRNAs in resting microglia, however, no significant difference was observed in Igf2bp2 expression levels between resting and activated microglia. Our results imply that, although microglia are considered as macrophages in the brain, these two types of cells might not share the same mechanisms in m6A modifications. Therefore, the influence of Igf2bp2 on microglial m6A modifications may be much minor than that of Igf2bp1.

Our study further revealed that Igf2bp1 modulates microglial activation *via* promoting the m6A methylation and stability of *Gbp11* and *Cp* mRNAs. Cp is an enzyme containing six copper atoms with important functions in iron homeostasis and inflammation (23, 24). Cp has been found to express in activated microglia (24). Cp enhances NO production, facilitates pro-inflammatory and neurotoxic mediator expression, and activates MAPK and NF-κB signaling pathways, which, in turns, activates BV2 microglial cells (25). Our results match with these literatures that suggest Cp as a pro-inflammatory factor. In contrast, the roles of Gbp11 in inflammation remain unknown. It has been reported that, in lung and livers, Gbp11 expression is induced by pro-inflammatory factors including LPS and IFN-γ (26, 27). We are the first group that demonstrated Gbp11 as a microglial activation regulator. However, whether Gbp11 is also involved in inflammatory response modulation in other types of immune cells requires to be examined. Importantly, it is reported that Igf2bp1 and Igf2bp2 mainly bind to mRNA 3’UTRs, while Igf2bp3 predominantly binds to the coding region of mRNAs (28). MeRIP-seq analyses in our study demonstrated that the m6A peaks of both *Gbp11* and *Cp* mRNAs were also located in the 3’UTR. Hence, our results imply that Igf2bp1 enhances the m6A methylation and mRNA stability *via* binding to the 3’UTRs of its targets.

In summary, our study has demonstrated a positive correlation between m6A modification patterns and mRNA expression in LPS-stimulated microglia, which is under the regulation of m6A reader Igf2bp1. We further demonstrated that Igf2bp1 regulates the inflammatory responses of microglia presumably *via* enhancing m6A methylation and stability of *Gbp11* and *Cp* mRNAs. Thus, our study provides a possible mechanism for the m6A methylation-mediated microglia regulation and identifies Igf2bp1 as a potential target for alleviating microglia hyperactivation.

## Data Availability Statement

The original contributions presented in the study are publicly available. This data can be found here: ArrayExpress, the accession numbers: E-MTAB-11568; E-MTAB-11411.

## Ethics Statement

The animal study was reviewed and approved by Institutional Animal Care and Use Committee (IACUC) of Tongji University School of Medicine.

## Author Contributions

XX, JZ conceptualized the project and designed the experiments. LD, HW, YL, ZL performed the experiments. LD, HW, XX, YW analyzed the data. XX wrote the manuscript. All authors reviewed the manuscript. All authors contributed to the article and approved the submitted version.

## Funding

This work was supported in part by research grants from the National Natural Science Foundation of China (No. 91949204 and No. 81830037 to JZ, No. 81971145 and No. 81901333 to XX).

## Conflict of Interest

The authors declare that the research was conducted in the absence of any commercial or financial relationships that could be construed as a potential conflict of interest.

## Publisher’s Note

All claims expressed in this article are solely those of the authors and do not necessarily represent those of their affiliated organizations, or those of the publisher, the editors and the reviewers. Any product that may be evaluated in this article, or claim that may be made by its manufacturer, is not guaranteed or endorsed by the publisher.

## Abbreviations

CNS, Central nervous system; CP, Ceruloplasmin; DAVID, Database for Annotation, Visualization and Integrated Discovery; DEG, Differentially expressed genes; ELISA, Enzyme-Linked Immunosorbent Assay; FPKM, Fragments per kilobase of transcript per million fragments mapped; Gbp11, Guanylate-binding protein 11; GO, Gene ontology; Igf2bp, Insulin-like growth factor 2 mRNA binding protein; KEGG, Kyoto Encyclopedia of Genes and Genomes; LOF, Loss-of-function; LPS, Lipopolysaccharide; MeRIP-seq, Methylated RNA immunoprecipitation sequencing; qRT-PCR, Quantitative Reverse Transcription Polymerase Chain Reaction; RNA-seq, RNA sequencing; SDS-PAGE, Sodium dodecyl sulfate polyacrylamide gel electrophoresis; UTRs, Untranslated Regions.
