# Supplementary figures and images for: m6A Reader Igf2bp1 Regulates the Inflammatory Responses of Microglia by Stabilizing Gbp11 and Cp mRNAs
Source: Front Immunol. 2022 Apr 29;13:872252. doi: 10.3389/fimmu.2022.872252 (PMC9100696; doi:10.3389/fimmu.2022.872252)

igf2bp1+marker

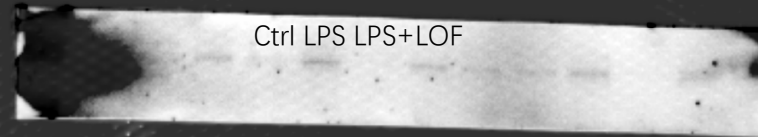

Actin+marker

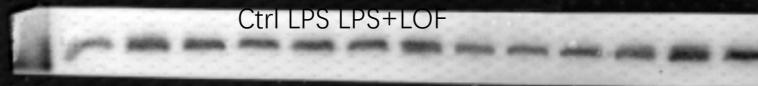

Supplement: Supplementary file 1 [file DataSheet_1.pdf]
